# Use of Positron Emission Tomography for Real-Time Imaging of Biodistribution of Green Tea Catechin

**DOI:** 10.1371/journal.pone.0085520

**Published:** 2014-02-03

**Authors:** Kosuke Shimizu, Tomohiro Asakawa, Norihiro Harada, Dai Fukumoto, Hideo Tsukada, Tomohiro Asai, Shizuo Yamada, Toshiyuki Kan, Naoto Oku

**Affiliations:** 1 Department of Medical Biochemistry, School of Pharmaceutical Sciences, University of Shizuoka, Shizuoka, Shizuoka, Japan; 2 Department of Synthetic Organic & Medicinal Chemistry, School of Pharmaceutical Sciences, University of Shizuoka, Shizuoka, Shizuoka, Japan; 3 Central Research Laboratory, Hamamatsu Photonics K.K., Hamamatsu, Shizuoka, Japan; 4 Department of Pharmacokinetics & Pharmacodynamics, School of Pharmaceutical Sciences, University of Shizuoka, Shizuoka, Shizuoka, Japan; NIH, United States of America

## Abstract

The aim of this study was to achieve real-time imaging of the in vivo behavior of a green tea polyphenol, catechin, by positron emission tomography (PET). Positron-labeled 4″ -[^11^C]methyl-epigallocatechin gallate ([^11^C]Me-EGCG) was orally administered to rats, and its biodistribution was imaged for 60 min by using a small animal PET system. As the result, images of [^11^C]Me-EGCG passing through the stomach into the small intestines were observed; and a portion of it was quantitatively detected in the liver. On the other hand, intravenous injection of [^11^C]Me-EGCG resulted in a temporal accumulation of the labeled catechin in the liver, after which almost all of it was transferred to the small intestines within 60 min. In the present study, we succeeded in obtaining real-time imaging of the absorption and biodistribution of [^11^C]Me-EGCG with a PET system.

## Introduction

A number of previous studies have provided evidence that the intake of green tea is associated with various biological activities such as anti-obesity, anti-oxidative, and anti-viral ones, as well as others [1], [2]. Among the components of green tea, catechins, which are polyphenols, play an important role in these activities, and many mechanistic studies on them have been performed. Especially, the relationship between (–)-epigallocatechin-3-*O*-gallate (EGCG), a major component of green tea catechins, and the anti-cancer effect of green tea is very significant, since cancer is the leading cause of human death in various countries. A previous study of ours demonstrated that EGCG suppresses tumor growth through the inhibition of membrane type I metallomatrix proteinase (MT1-MMP) activity and subsequent tumor angiogenesis [3]. Tachibana *et al*. further proposed a receptor of EGCG to be laminin 5, as they demonstrated the direct binding of green tea catechin to this molecule [4]–[6]. These findings indicate that EGCG might well be a functional component for cancer therapy and chemoprevention. However, there is little information about the biodistribution of catechins after their intake; because the synthesis of artificial EGCG is quite complicated, and the detection of catechins or their metabolites in the body is difficult. On the other hand, we recently succeeded in developing a novel method for the synthesis of EGCG and its derivatives, and investigated the intracellular distribution of this synthetic EGCG in human umbilical vein endothelial cells (HUVEC) by using fluorescence-labeled EGCG [7]. The results indicated that a portion of this EGCG accumulates in the mitochondria of these cells, suggesting that mitochondrial dysfunction might be involved in the anti-angiogenic effect of EGCG [7]. Such an approach using polyphenol derivatives conjugated to a functional moiety is useful for the elucidation of the mechanism of EGCG function.

Imaging technologies for determining the whole-body distribution of bioactive molecules and drugs are now growing, and various devices for such imaging have been developed. Positron emission tomography (PET) is a noninvasive-imaging technique and has been used for clinical diagnosis in many fields such as oncology and neurology. This technique can also be applied in biodistribution studies performed for the development of novel drugs, referred to as “phase 0” studies, since PET can acquire real-time 3-dimensional image data of the biodistribution of a positron-labeled agent in the whole body by the injection of a microgram dose of it. PET data obtained in a phase 0 study can be used to predict unexpected side effects of an agent in development, predictions based on unfavorable pharmacokinetics, before the clinical study is performed. With this use in mind, we previously synthesized positron-labeled small-interfering RNA (siRNA) and were successful in imaging the systemic biodistribution of it after intravenous injection into rodents [8]. We had already proposed a novel positron-labeling method for following preformed lipid nanoparticles and demonstrated by PET imaging that liposomes bearing encapsulated hemoglobin accumulate in an ischemic region of the rat brain [9]. Gulyás *et al*. have already succeeded to image the biodistribution of [^11^C]vinpocetine after oral administration to a human and demonstrated its permeability of blood-brain barrier [10]. In light of such studies, we considered that PET scanning would be absolutely suitable for whole-body imaging of trace amounts of food components such as green tea catechins. Actually, we have already synthesized positron-labeled nobiletin, a polymethoxylated citrus flavone, and succeeded in imaging the whole-body biodistribution of it by using PET and showing its temporal accumulation in the brain after intravenous injection [11].

In the present study, to image the biodistribution of green tea catechin, we synthesized a positron-labeled catechin, 4″-[^11^C]methyl-epigallocatechin gallate ([^11^C]Me-EGCG), and monitored the real-time trafficking of the orally administered catechin with a PET system. As another route, [^11^C]Me-EGCG was intravenously injected into rats via a tail vein; and the biodistribution obtained via blood circulation was evaluated.

## Materials and Methods

### Agents

*n*-Bu_4_NOH and DMSO (anhydrous) were purchased from Sigma-Aldrich Co. (St. Louis, MO, USA). High purity N_2_ gas (99.9999%) was obtained from Japan Fine Products (Kawasaki, Japan). LiAlH_4_ was from ABX GmbH (Redeberg, Germany); and 57% hydroiodic acid, from Nacalai Tesque, Inc. (Kyoto, Japan). All other reagents were commercially available and used without purification unless otherwise stated.

### Animals

Six-week old Wistar male rats were purchased from Japan SLC Inc. (Shizuoka, Japan). All animals used in this study were fed and handled subject to the guidelines of University of Shizuoka and those of the Central Research Laboratory, Hamamatsu Photonics K.K. All animal procedures were approved by the Animal and Ethics Review Committee of University of Shizuoka and that of the Central Research Laboratory, Hamamatsu Photonics K.K.

### Synthesis of [^12^C]Me-EGCG for the Authentic Standard

**Figure 1:**
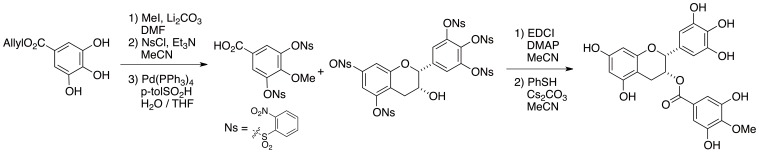
Synthesis of [^12^C]Me-EGCG for the authentic standard. DMF = *N,N*-Dimethylformamide, THF = Tetrahydrofuran, NsCl = *o*-Nitrobenzensulfonyl chloride, EDCI = 1-Ethyl-3-(3-dimethyl aminopropyl)carbodiimide, DMAP = *N,N*-Dimethyl-4-aminopyridine.

The synthesis of various methylated catechins including 4″-Me-EGCG by utilizing our original synthetic method and the investigation of their biological activities in comparison with EGCG have been reported by us [12]. Since the sample of [^12^C]Me-EGCG could be in a gram scale as shown in Figure 1, HPLC profile has been determined by employment it as an authentic sample.

### Synthesis of Positron-labeled Catechin

[^11^C]CO_2_ was produced by proton bombardment (18 MeV, 20 µA) of N_2_ by using a cyclotron (HM-18, Sumitomo Heavy Industries, Tokyo, Japan) at Hamamatsu Photonics PET center. [^11^C]CO_2_ in the target chamber was recovered with N_2_ and bubbled through 0.1 M LiAlH_4_ in THF. After evaporation of the THF with a N_2_ flow, the resulting LiAl(O[^11^C]CH_3_)_4_ was converted to [^11^C]CH_3_I by treatment with 57% hydroiodic acid (HI) [13]. Gaseous [^11^C]CH_3_I was then transferred to a solution of EGCG (4 mg, 8.7 µmol) and 1 M *n*-Bu_4_NOH (CH_3_OH solution, 0.87 µL, 0.87 µmol, 0.1 Eq) in DMSO (0.3 mL). This mixture was reacted for 3 min at 80°C followed by cooling to room temperature. The reaction mixture was purified by HPLC (column, Inertsil ODS3, 7.6×250 mm; mobile phase, CH_3_CN/H_2_O/CH_3_COOH/100 mg/mL ascorbic acid = 150/850/2/1; flow rate, 6 mL/min; detection wavelength, 280 nm). The radioactive peak corresponding to an authentic sample (12.6 min) was collected, and the mobile phase was evaporated under reduced pressure. The residue was dissolved in saline and delivered into a sterile vial through a 0.22-µm pore membrane filter (Milipore, Billerica, MA, USA). An aliquot of the final product was analyzed by HPLC (column, Inertsil ODS3, 4.6×150 mm; mobile phase, CH_3_CN/H_2_O/TFA = 200/800/1; flow rate, 1 mL/min; detection wavelength, 280 nm). The result indicated that 4″-methoxy EGCG, termed [^11^C]Me-EGCG, was specifically yielded, as determined by HPLC (data not shown). The yield, radiochemical yield, radiochemical purity, and specific activity were 0.56 GBq, 2.8%, 100%, and 30.3 GBq/µmol (n = 5), respectively.

### Preparation of Sample Solution

Synthesized [^11^C]Me-EGCG was diluted with saline to adjust the radioactivity to approx. 25 MBq/animal for injection. The radioactivity of the injected sample was calculated by measuring the radioactivity with a curiemeter (IGC-7, Aloka, Japan) before and after injection and further correcting under consideration of the half-life of ^11^C and the emission time.

### PET Study

The biodistribution of [^11^C]Me-EGCG was imaged noninvasively by using a small-animal PET system. In brief, a Wistar rat (141.2 g) anesthetized with chloral hydrate (50 mg/mL, 0.2 mL/h) was fixed on the stage of a Clairvivo PET device (Shimadzu, Japan), which was then placed on the device for measuring the background. After the transmission, the stage bearing the rat was removed; and [^11^C]Me-EGCG solution (17.7 MBq; 0.5 mL) was orally administered to the animal via a stainless steel probe. Immediately thereafter, the stage was replaced on the PET apparatus; and the PET scan was started from 1 min after sample injection. After a 60-min PET scan, the rat was placed on an animal CT (Clairvivo CT, Shimadzu, Japan) to obtain the CT images.

For a PET imaging study on the biodistribution after a systemic injection, [^11^C]Me-EGCG solution (14.3 MBq; 0.5 mL) was intravenously injected into a rat (143.8 g) via a tail vein. The PET scan was started immediately after the administration of [^11^C]Me-EGCG and continued for 60 min.

### Quantitative Analysis of [^11^C]Me-EGCG Accumulation in Organs

In a separate study from PET scan, the accumulation of [^11^C]Me-EGCG in the rat organs was investigated by a conventional method. In brief, [^11^C]Me-EGCG solution (9.61±1.56 MBq; 0.5 mL) was orally administered to rats (n = 4; mean weight: 145.6 g). After 60 min, the rats were sacrificed under anesthesia; and the blood and organs (heart, liver spleen, brain, stomach, and small intestine) were then harvested. Feces in the small intestines was removed as much as possible. Then, the blood and organs were weighed; and the radioactivity was measured by a use of a γ-counter (1480 Wizard 3, PerkinElmer, USA). The raw data were corrected for disintegration of radioactivity by considering both the half-life of ^11^C and the time from administration time to measurement time. The distribution data were presented as the percentage of injected dose per whole tissue. The total weight of blood was assumed to be 7.56% of the body weight.

For the biodistribution study with systemic injection, [^11^C]Me-EGCG solution (0.57±0.15 MBq: 5 min, 1.16±0.18 MBq: 60 min; 0.5 mL) was intravenously injected via a tail vein (n = 4), and the accumulation in each organ (heart, liver spleen, and brain) after 5 and 60 min was determined similarly as described above.

## Results and Discussion

### Synthesis of [^11^C]Me-EGCG

**Figure 2:**
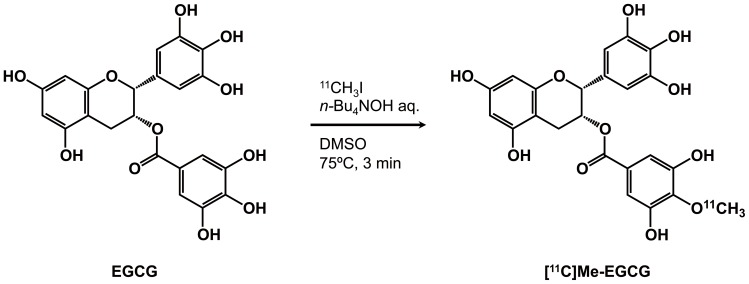
Reaction for synthesis of [11C]Me-EGCG.

To examine the accurate biodistribution of green tea catechins and to image the real-time behavior of them after oral intake, we synthesized positron-labeled catechin and analyzed its biodistribution with a PET. For obtaining positron-labeled catechin, a one-step, simple synthesis methodology is essential due to the short half-life of positron-emitting nucleotides. In addition, it is not recommended to introduce any other functional moiety to the target compound used for positron labeling because of possible alteration of the original characteristics of the compound. With these considerations in mind, we introduced a [^11^C]methoxy moiety to the gallate group of EGCG to obtain positron-labeled catechin, since it is well known that there are some methoxy catechins in green tea. As shown in Figure 1, by the treatment of EGCG with [^12^C]MeI under a basic condition, the regioselective alkylation reaction proceeded at 4″ position of EGCG to give the [^12^C]Me-EGCG. The confirmation of the methylated position was performed by HMBC analysis. Since the most reactive hydroxyl group is *para* position of carbonyl moiety among the eight phenols of EGCG, this methylation proceeded specifically at 4″-position. On the other hand, we previously compared MMP inhibitory activity of methylated EGCGs with EGCG and demonstrated that the activity of 4″-Me-EGCG was a little bit weakened in comparison with EGCG, but 4″-Me-EGCG sufficiently possessed the potentials in similar to EGCG [12]. As shown in Figure 2, the synthesis of positron-labeled EGCG was accomplished by similar procedure for the preparation of [^12^C]Me-EGCG. The completion of the alkylation reaction required 3 min, and the purification was finished within 20 min. Actually the structure of [^11^C]Me-EGCG was identified by comparison with the our synthetic [^12^C]Me-EGCG and the peaks detected by UV and radioactivity in HPLC chromatograms were overlapped. Although the predominant decreasing of [^11^C]Me-EGCG was observed in the usual HPLC purification conditions, the addition of ascorbic acid to the developing solvent played a key role for preventing from the decomposition reactions. In this way, the rapid as well as efficient synthetic method of [^11^C]Me-EGCG for PET study has been established.

### PET Analysis of Biodistribution of [^11^C]Me-EGCG

**Figure 3:**
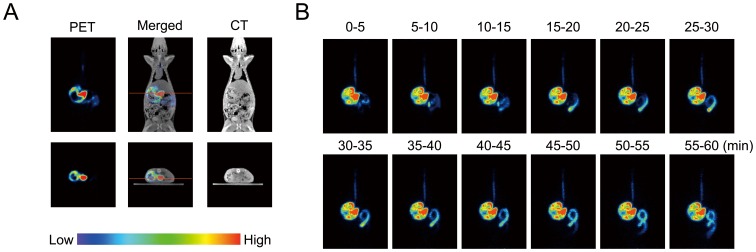
Whole-body PET imaging of distribution of [^11^C]Me-EGCG after oral administration. [^11^C]Me-EGCG solution was orally administered to a Wistar rat. PET scanning was started from 1 min after the administration and continued for 60 min. (A) Horizontal (upper panel) and vertical (lower panel) cross-section images were obtained from the data accumulated during 56–61 min after the administration. The red line indicates the position of the cross section. (B) Photographs indicate the horizontal images integrated every 5 min.

**Figure 4:**
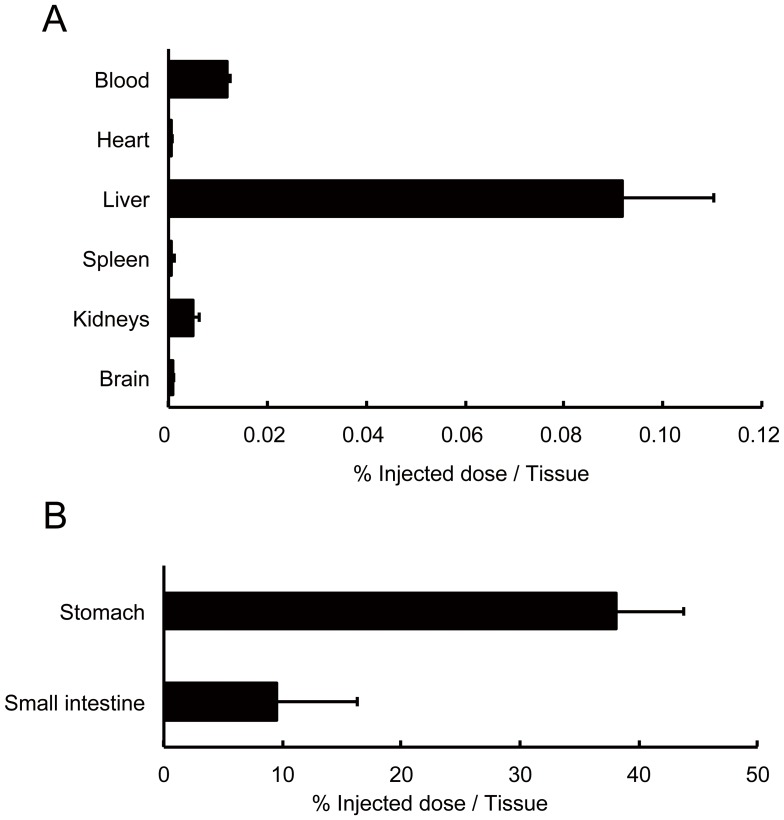
Accumulation of [^11^C]Me-EGCG in the organs after oral administration. [^11^C]Me-EGCG solution was orally administered to Wistar rats (n = 4); and the accumulation of ^11^C in blood, heart, liver, kidneys, and brain (A), and in stomach and small intestine (B) at 60 min after administration was determined by using a γ-counter. Data are presented as the percent of the injected dose per whole tissue and S.D.

To determine the biodistribution of the imaged green tea catechin, we first scanned the rat for 60 min with the PET system after oral intake of [^11^C]Me-EGCG. As the result, real-time images of [^11^C]Me-EGCG distribution in the body were clearly observed; and both vertical and horizontal images were obtained (Figure 3A). This whole-body imaging analysis of [^11^C]Me-EGCG over time revealed that most of the [^11^C]Me-EGCG was first detected in the stomach and that a portion of it then moved into the intestines during the scanning (Figure 3B). This result was also supported by the data obtained with a γ-counter, showing that most of the ^11^C radioactivity was detected in the stomach and small intestines (Figure 4). Furthermore, small but certain amounts of [^11^C]Me-EGCG were found in the blood (0.012%) and liver (0.092%) at 60 min after administration. Earlier, Chen *et al.* demonstrated that catechins having a galloyl moiety, such as EGCG, have a longer blood circulation time after intravenous injection of catechins into rats than catechins without this moiety, such as EGC and EC [14]. By using UPLC/ESI-MS, Misaka *et al.* previously examined the pharmacokinetics of some catechins after oral intake of green tea polyphenols and demonstrated that these catechins could be detected in the bloodstream as their intact forms up to even 24 h after intake and that the concentration of EGCG in the bloodstream was especially higher than that of other catechins [15]. These findings strongly support our contention that the present imaging data obtained with ^11^C adequately reflected the [^11^C]Me-EGCG behavior in the body.

**Figure 5:**
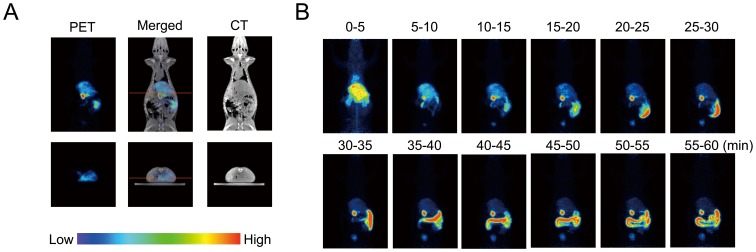
PET imaging of biodistribution of [^11^C]Me-EGCG after intravenous injection. Biodistribution of [^11^C]Me-EGCG was determined with a small-animal PET system (Clairvivo PET). [^11^C]Me-EGCG solution was intravenously injected to a Wistar rat via a tail vein, and PET scanning was started immediately after the injection. (A) Photographs indicate horizontal (upper panel) and vertical (lower panel) images obtained from the data accumulated during 10–15 min. (B) The data were acquired for 60 min and integrated every 5 min.

**Figure 6:**
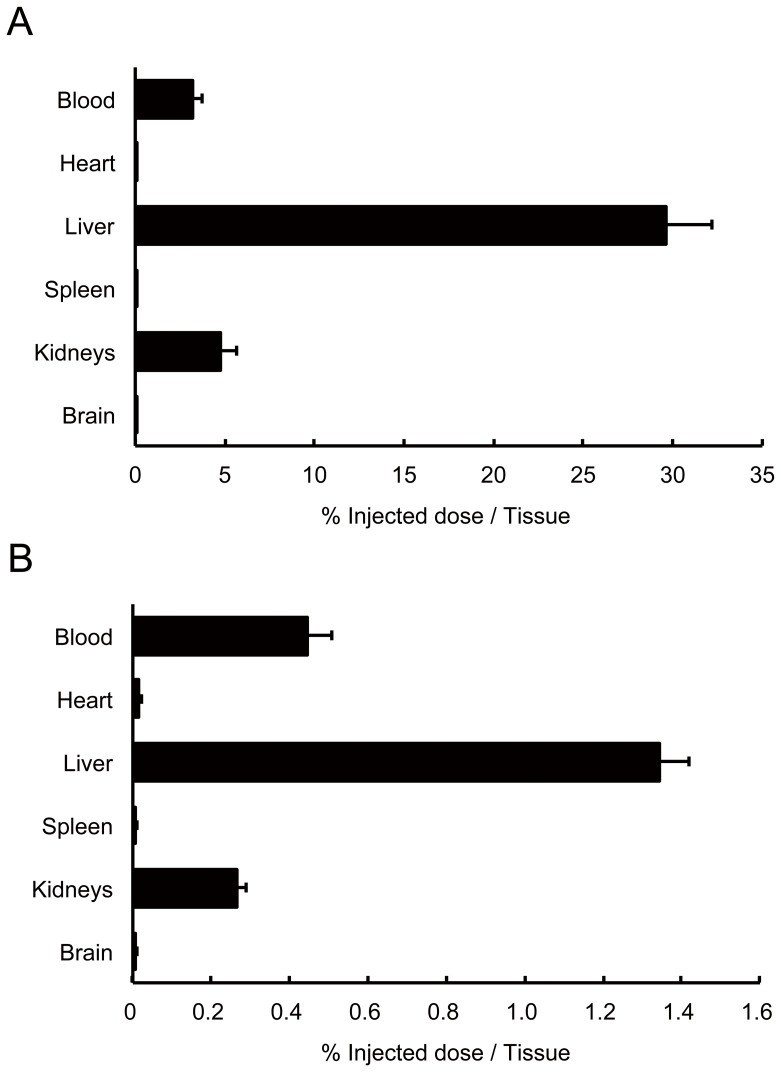
Biodistribution of [^11^C]Me-EGCG after intravenous injection. [^11^C]Me-EGCG solution was intravenously injected into Wistar rats (n = 4) via a tail vein, and the accumulation of ^11^C in each organ at 5 min (A) and 60 min (B) after the injection was measured by using a γ-counter. Data are presented as the percent of the injected dose per whole tissue and S.D.

To mimic the behavior of EGCG in the bloodstream and to image the biodistribution via this route, we injected [^11^C]Me-EGCG intravenously into rats and scanned the behavior in real time by PET. The results of the imaging analysis revealed that [^11^C]Me-EGCG temporarily accumulated in the liver after the injection (Figure 5A). When the accumulation of [^11^C]Me-EGCG in each organ was quantified by measuring the radioactivity of ^11^C, most of the [^11^C]Me-EGCG had actually accumulated in the liver at 5 min, and the accumulation thereafter decreased at 60 min (Figure 6). The blood concentration of [^11^C]Me-EGCG was about 4% of the injected dose at 5 min and was reduced to one-tenth of that by 60 min. It is remarkable that the accumulation profile for the organs was very similar between the oral administration and intravenous injection, although the actual amounts of in each organ were different. In the case of the oral administration, the accumulation in the liver was higher than that in the other organs except the stomach and small intestines. Thus, these biodistribution data reflected the behavior of [^11^C]Me-EGCG after its transference to the bloodstream. On the other hand, after temporary accumulation in the liver following the intravenous injection, [^11^C]Me-EGCG readily started to be transferred to the small intestines; and by 60 min after the injection, most of it had been transferred out of the liver, and a slight amount was detected in the bladder (Figure 5B). Thus, this result suggested that [^11^C]Me-EGCG was metabolized in the liver and excreted into either the small intestines via the bile or the urine via the kidneys. Actually, Lambert *et al.* earlier reported that EGCG is glucuronidated in the liver after oral administration to mice and mainly excreted in the feces [16]. Lu *et al.* also demonstrated, using rat liver cytosolic enzyme, that EGCG is easily methylated by catechol-*O*-methyltransferase (COMT) or glucuronidated by UDP-glucuronosyltransferase (UGT) on either the B-ring or D-ring of its structure and thereby converted to methylated or glucuronidated catechins such as 4″-*O*-methyl-EGCG, 4′,4″-di-*O*-methyl-EGCG, and EGCG-4″-*O*-glucuronide [17], [18]. These findings further support our data that [^11^C]Me-EGCG accumulated in the liver after circulation in the bloodstream, was metabolized there, and was finally secreted into the small intestines.

## Conclusions

In the present study, we succeeded to synthesize the positron-labeled catechin and imaged the whole-body biodistribution of our positron-labeled catechin by PET; and the real-time imaging data showed that almost all of the orally-administered green tea catechin was retained in the stomach until 60 min, but that a portion of it was absorbed into the bloodstream from the intestines, temporally accumulated in the liver, and then returned to the small intestines. This present study is the first to have imaged the biodistribution of green tea catechin with PET and provides valuable information for elucidating the bioactivities of green tea catechin and its potential as a functional food component. PET technology is now growing and is being widely applied in medical fields for purposes such as disease diagnosis and drug development. Therefore, we believe that its continued use will contribute to the further improvement of human health and increased longevity.
